# Zika Virus: A Review of Literature

**DOI:** 10.7759/cureus.3025

**Published:** 2018-07-22

**Authors:** Vivian C Agumadu, Kamleshun Ramphul

**Affiliations:** 1 Medicine, International University of the Health Sciences School of Medicine, Basseterre, KNA; 2 Pediatrics, Shanghai Jiao Tong University School of Medicine/Shanghai Xin Hua Hospital, Shanghai, CHN

**Keywords:** zika virus, microcephaly, aedes mosquito

## Abstract

Zika virus (ZIKV) is an arthropod-borne virus that was first isolated in 1947. Since then, multiple outbreaks of ZIKV have been reported in different countries. It is transmitted mostly by Aedes mosquitoes, and the symptoms of fever, joint pain, red eyes, headache, and maculopapular rash closely resemble chikungunya and dengue. The most severe complications of ZIKV infection include the risk of microcephaly and other congenital brain anomalies in infected pregnant women. It is a condition that can be easily prevented with proper education. In this review, we focussed on different aspects of the recent outbreaks of Zika virus disease, including its epidemiological background, transmission routes, presentation, as well as diagnosis and prevention.

## Introduction and background

Zika virus (ZIKV) is an arbovirus that was first isolated from a rhesus sentinel monkey in Kampala, Uganda, in 1947 [1]. The first case of infection in humans was described in a 10-year-old female from Nigeria in 1954 [2]. In 2007, the first major outbreak of Zika fever took place on the Western Pacific Island of Yap in the Federated States of Micronesia [3]. Since then, multiple outbreaks of ZIKV have been reported worldwide. Two larger epidemics affecting over 30,000 people took place in 2013 and 2014 in French Polynesia [4-5]. Smaller outbreaks also cropped up in different Pacific Islands, such as New Caledonia, the Easter Island, and the Cook Islands, along with the Solomon Islands, Samoa, and Vanuatu. The condition slowly crawled its way to the Americas in March 2015, and at the end of January 2016, more than 20 countries reported outbreaks of the ZIKV [6-7].

The main complication of Zika virus infection is the risk of microcephaly and other congenital brain anomalies in infected pregnant women. The World Health Organization (WHO) raised the alarm for this condition, as most cases of microcephaly and defects in newborns from Zika virus disease were noted in economically deprived countries. The organization emphasized that it is vital to properly educate the public on preventive measures to avoid a similar occurrence in the future.

## Review

Method

We conducted a review of the different aspects of the recent outbreaks of Zika virus disease, including an epidemiological study, an investigation of the transmission routes, the signs and symptoms of the disease, as well as the diagnosis and prevention of Zika virus disease. A search was executed through PubMed and Google Scholar. The keywords used were: “Zika virus disease,” “Epidemiology of Zika virus disease,” “mortality and morbidity of Zika virus disease,” “transmission of Zika virus disease,” “Prevention of Zika virus disease,” and “Pregnancy and Zika virus disease.” The timeline was set from January 1950 to January 2018.

What is Zika virus?

ZIKV is described as an arthropod-borne virus that belongs to the Flaviviridae family consisting of the Flavivirus genus and Hepacivirus genus. The former consists of Zika virus, yellow fever virus, West Nile virus, and dengue virus, while hepatitis C virus forms part of the Hepacivirus genus. It is a positive, single-stranded ribonucleic acid (RNA) virus with an envelope making it closely related to the Spondweni virus [7]. Two main lineages have been reported: the African lineage and the Asian lineage [8].

How is Zika virus transmitted?

Zika virus has different modes of transmission. The Aedes mosquito bite is the most common one, and it involves different families of Aedes: A. aegypti, A. albopictus, A. polynesiensis, A. vittatus, A. unilineatus, and A. hensilli among many other previously reported subtypes [9]. Vertical transmission from an infected mother through the placenta to the fetus has also been previously reported. Furthermore, Zika virus can be transmitted through blood transfusions and through sexual encounters, whether oral, vaginal, or anal via an infected person [10]. In 2016, the first male-to-male transmission was also registered in Texas, US. However, the major and most serious threat of transmission is mosquito bites [11].

What is the pathogenesis of Zika virus?

Zika virus most commonly follows the sylvatic transmission cycle. It is classified as an arbovirus that is transmitted through one vertebrate to another by a mosquito bite. The virions exist as immature (non-infectious), mature (infectious), and fusogenic (host membrane binding) states. Humans are incidental hosts in the lifecycle of the virions. Monkeys and apes can also serve as host to the virions, while some studies have found that sheep, elephants, and goats had antibodies against Zika, suggesting possible host states as well [12].

The cycle starts when an Aedes mosquito ingests blood containing Zika virus after biting an infected person. The virus starts replicating in the epithelial cells of midgut and goes to the salivary glands of the mosquito. After an incubation period of 10 days, the saliva becomes infected, making the mosquito a vector for infecting a human. Upon entry into the human skin, the virus infects the dermal fibroblasts that serve as receptors for attachment of Zika virus [13]. Upregulation of TLR3 mRNA expression is triggered and this leads to an enhanced innate immune response. Interferon alpha and beta are produced by the infected cells to help lower the viral load. Zika virus can also lead to the autophagy of host cells and inhibitors to this step can be a potential treatment option in the future. Once replication is complete, the virus spreads to the regional lymph nodes hematogenously, contaminating distant organs such as the nervous system on its way. Moreover, in pregnancy, the virus can go through the placental blood barrier to infect the fetus [9,11,14-15].

What are the clinical symptoms of Zika virus disease?

Misdiagnosis of Zika virus disease is very common because signs and symptoms in previously healthy patients resemble chikungunya and dengue. After an incubation of three to 12 days, Zika fever appears [16]. The most commonly reported symptoms include fever, joint pain, red eyes, headache, and a maculopapular rash [17]. The symptoms usually last less than a week and no mortality has yet been reported from the initial infection phase.

The complications of Zika virus in pregnant women include microcephaly and other brain malformations [18-19]. Infections in previously healthy adults have been associated with Guillain-Barre syndrome (GBS) as well [20].

How to diagnose and treat Zika virus disease?

Diagnosis of Zika virus disease relies mostly on the resources available in a country. The classic presentations and the exposure via either residence in or travel to a high-risk area or even contact with an infected person should raise suspicions. The Centers for Disease Control and Prevention (CDC) highly recommends testing for Zika virus in patients who had recent exposure and have presented with recent symptoms of Zika, pregnant women with possible Zika virus exposure, asymptomatic pregnant women currently exposed to Zika virus, pregnant woman with possible Zika virus exposure having been diagnosed with possible congenital Zika virus infection via prenatal ultrasound and for asymptomatic pregnant woman with no ongoing but recent exposure to Zika. However, Zika virus testing is not recommended as preconception screening or in non-pregnant asymptomatic individuals.

Other recommendations by the CDC are as below:

Any non-pregnant symptomatic patient should undergo a serum and urine testing for Zika virus ribonucleic acid (RNA), nucleic acid testing (NAT) and Zika virus and dengue virus immunoglobulin M (IgM) testing in serum. NAT is most useful when samples are collected within two weeks of symptom onset. Zika virus and dengue virus IgM should also be tested when the NAT sample collection is done. The IgM levels can be tested as well after 14 days of symptom onset while NAT is not recommended after two weeks of symptom onset.

For a symptomatic pregnant woman, it is advised to test for serum and urine NAT and Zika virus IgM in serum up to 12 weeks of symptom onset. If the patient’s serum and urine NAT are positive, then it should be considered as an acute maternal Zika virus infection even if the IgM result is negative. If either serum or urine NAT is positive but not both, and Zika IgM is positive, it should be diagnosed as acute Zika virus infection. If both serum and urine NAT are negative and IgM is positive, a plaque reduction neutralization test (PRNT) should be performed on the patient.

Any asymptomatic pregnant woman with current Zika virus exposure has been tested during pregnancy. NAT testing ought to be done during her first initial prenatal visit, followed by two more testing during pregnancy with two non-consecutive visits. IgM serology is not useful and not routinely recommended especially if the patient has previously been confirmed for Zika virus infection. If any of the NAT tests is positive during pregnancy, no further NAT testing should be done.

If a woman is asymptomatic and pregnant and was recently exposed to Zika virus but currently not in direct exposure, testing should be considered only on a case-by-case basis and the gap between the last exposures should be considered. Any pregnant woman with possible exposure to Zika and diagnosed with congenital Zika virus infection via prenatal ultrasound should be tested for NAT and IgM on serum and urine as described for symptomatic pregnant women. More invasive procedures and testing such as amniocentesis, placental, and fetal tissues testing can be considered if required [21].

The current treatment protocol for Zika virus infection involves mostly symptomatic care. Patients are advised to have proper bed rest and maintain hydration. Any pain or fever can be treated via acetaminophen. It is also recommended to avoid aspirin or other non-steroidal anti-inflammatory drugs before dengue is properly ruled out due to increased risk of bleeding associated with the dengue [22].

How to prevent the spread of Zika virus?

Since Zika virus is spread mostly by the Aedes mosquitoes, preventive measures should focus on eliminating them. The mosquitoes bite during both the day and night, therefore, the use of mosquito repellents that are safe and effective for pregnant, breastfeeding women, or even the elderly should be considered. If someone is using a sunscreen, it is advised to first apply the sunscreen and then apply the mosquito repellent. The application of mosquito repellents in babies younger than two months of age is not advised. It is also advised to avoid applying any repellent close to any child’s eyes or mouth. Furthermore, the children should be taught to avoid touching their eyes or mouth after being sprayed on a particular body part. The use of oil of lemon eucalyptus (OLE) or para-menthane-diol (PMD) is not advised for children younger than three. Other preventive measures include the proper use of mosquito nettings and long-sleeved shirts and long pants whenever possible. At home, it is advised to prevent mosquitoes from breeding by getting rid of any items that can be a source of stagnant water such as flowerpots, trash containers, and tires. It is also recommended to avoid sleeping outdoors and limit outdoor activities [21,23].

Any plans to travel to high-risk areas during pregnancy should be postponed [24]. Since Zika virus can also be transmitted via semen and vaginal fluid of infected people, abstinence till medically cleared should be encouraged. Condoms have been found to reduce the risk of spreading Zika during intercourse and for more-effective impact, it should be used throughout the intercourse [25]. Anyone caring for an infected person has to avoid touching any bodily fluids or blood. Proper handwashing techniques using soap and water should be done regularly. Clothes with blood or bodily fluids ought to be washed using proper detergents and temperatures. The use of bleach is not required for these patients.

The key to the prevention of Zika virus infection in underdeveloped countries involves mostly the proper education of the public. At present, there are no vaccines or prophylactic treatments available for the disease. While several mice studies have shown promising results, it will take a few more years before one is available safely. Until then, most countries should focus on prevention, early detection in pregnant woman and proper management, as well as contact tracing to lower contamination risk, and prevent an outbreak in the future.

## Conclusions

While Zika virus is not deadly, the complications associated with it, especially in pregnant women and children, can compromise quality of life. It can easily be prevented with proper measures and health management. With yearly outbreaks globally, all health groups should devise a proper plan ahead of peak season.
